# Editorial: Epidemic status and prevention of swine infectious diseases

**DOI:** 10.3389/fvets.2023.1169644

**Published:** 2023-03-06

**Authors:** Mo Zhou, Lianfeng Li, Keisuke Suganuma

**Affiliations:** ^1^Jiangsu Key Laboratory for High-Tech Research and Development of Veterinary Biopharmaceuticals, Engineering Technology Research Center for Modern Animal Science and Novel Veterinary Pharmaceutic Development, Jiangsu Agri-Animal Husbandry Vocational College, Taizhou, China; ^2^Harbin Veterinary Research Institute, Chinese Academy of Agricultural Sciences, Harbin, China; ^3^National Research Center for Protozoan Diseases, Obihiro University of Agriculture and Veterinary Medicine, Obihiro, Japan

**Keywords:** epidemic status, prevention, diagnosis, swine, infectious diseases

Swine infectious disease is an essential factor affecting the stable development of the swine industry and has caused severe economic losses to the breeding industry. The rapid expansion of the swine breeding scale has provided certain primary conditions for the high incidence and spread of swine infectious diseases, which have a severe impact on the development of the swine breeding industry. Therefore, it is necessary to pay special attention to swine infectious diseases, fully understand their epidemic characteristics, formulate scientific and reasonable prevention and control countermeasures, and form scientific and effective prevention and control of swine infectious diseases.

The objective of this Research Topic was to bring attention to the swine infectious disease and gather different contributions highlighting essential aspects of contagious swine disease, including the epidemic status, the diagnostic method and the vaccine development.

Pseudorabies virus (PRV) causes reproductive problems in sows and boars and a high mortality rate in piglets, which results in huge economic losses for the swine industry (1). Currently, the widespread porcine PRV belongs to genotype II, and the protection of the traditional vaccine against genotype I PRV has declined. And new mutated strains can cause human infection (2). Zhang et al. investigated the prevalence of the PRV in the Hebei Province of China between 2017 and 2018. Serum samples collected showed a 46.27% positive rate for PRV gE antibodies. A total of 11 PRV variants have been isolated, and all are highly homologous, clustered in a similar group as HSD-1/2019, which causes acute encephalitis in humans.

Porcine circovirus type 2 and type 3 (PCV2 and PCV3) are two critical viral pathogens in the swine industry that have a significant economic impact in the world (3, 4). Nan et al. investigated the prevalence and genetic diversity of PCV2 in the northern Guangdong Province of China. 51.38% (297/573) of samples tested positive for PCV2. Study strains belonged to three genotypes of PCV2: PCV2a, PCV2b, and PCV2d. In addition, Yang et al. investigated the prevalence and genetic diversity of PCV3 and PCV2 in the southwest region of China between 2020 and 2022. 26.46% of the samples were positive for PCV2 and 33.46% for PCV3. Coinfection rates were 5.75% in 2020 and 10.45% in 2022. PCV2d is the predominant PCV2 genotype and the PCV3 isolates with high nucleotide homology and three mutations in antibody recognition domains. Their genotyping, immunogenicity, and immune evasion will be further studied under the help of these studies.

Porcine reproductive and respiratory syndrome virus (PRRSV) causes significant financial losses to the swine industry. Base on ORF5, type 2 PRRSV strains can be classified into NADC30-like, QYYZ-like, VR2332-like, and JXA1-like strains (5, 6). An analysis of genetic variation was conducted by Li P. et al. on the isolated PRRSV ORF5 gene. NADC30-like PRRSV strains are still dominant in Shandong Province, while NADC34-like are starting to become more prevalent. Xu et al. obtained 24 QYYZ-like PRRSV isolates from central and southern provinces of China. Therefore, QYYZ-like strains were commonplace in central and south China and played a role in the PRRSV epidemic by providing recombinant fragments. In addition, a genome-wide analysis of four PRRSV isolates from a single farm in China was conducted by Liu et al. Two isolates had 150-aa deletions identical to the live attenuated virus vaccine strain, and the pathogenicity of these isolates was different. PRRSV genomes have evolved through recombination between field strains and vaccine strains.

The swine enteric coronavirus (SeCoV) severely threatens public health and global security and causes significant losses for the global swine industry. A total of four SeCoV viruses are known to cause swine acute diarrhea syndrome: transmissible gastroenteritis virus (TGEV), porcine epidemic diarrhea virus (PEDV), porcine delta coronavirus (PDCoV), and swine acute diarrhea syndrome coronavirus (7, 8). Li M. et al. gave a comprehensive overview of the effects of CoV on apoptosis, autophagy, and innate immunity have been studied, providing insight into the pathogenic mechanism of the virus. Autophagy is an intrinsic defense mechanism that mediates the autophagic elimination of viral components, but viruses have evolved various strategies to escape or subvert the antiviral effects of autophagy. The study of SeCoV and its interaction with the host is crucial for understanding the pathogenic mechanism of the virus and developing effective treatments and preventions. Furthermore, several areas of Shandong Province were investigated for PEDV infection by Shen et al. Positive rates of 37.5% were found for PEDV, and significant variations were found in the structure domain region of the S gene. The study provides valuable information on the molecular epidemiology of PEDV and helps prevent and control the disease.

The African swine fever virus (ASFV) has spread rapidly in China, resulting in significant economic losses (9). The simultaneous presence of ASFV genotypes I and II in China made field identification more challenging since both genotypes are capable of causing chronic infection and morbidity in pigs. In response to this, Cao et al. developed a duplex fluorescent quantitative PCR assay to distinguish ASFV genotypes I and II based on the B646L sequences. The assay has high specificity and is highly reproducible, and it is an essential tool for detecting ASFV differentially.

Haemophilus parasuis (HPS) is one of the major infectious diseases affecting the swine industry globally and causes significant economic losses. Vaccination is the primary method of preventing HPS infections (10, 11). Based on pan-genomic analysis of 121 strains and reverse vaccine design, Pang et al. developed a multiepitope vaccine against HPS. Based on pre-predicted epitopes in the outer membrane proteins of the HPS core genome, the vaccine construct showed high immunogenicity and high Toll-like receptor 2 binding affinity. According to *in silico* immune simulations, the vaccine elicited an effective immune response. Mouse polyclonal antibodies produced from the vaccine protein bind different serotypes and non-typable HPS *in vitro*. Multiepitope vaccines are promising candidates for HPS pan-prophylaxis.

These studies in this research reported the epidemic status of the PRV, PCV, PRRSV, and PEDV and the genetic basis of these pathogens. Furthermore, this Research Topic also reported the development of the novel diagnostic methods and vaccines against ASFV and HPS. This Research Topic will help us to control swine infectious diseases effectively.

## Author contributions

All authors listed have made a substantial, direct, and intellectual contribution to the work and approved it for publication.
